# Hepatitis B Virus Reactivation Potential Risk Factors in Hepatocellular Carcinoma via Transcatheter Arterial Chemoembolization: A Retrospective Research

**DOI:** 10.1155/2021/8864655

**Published:** 2021-01-08

**Authors:** Xiaoguang Wang, Xiaodan Yang, Fei Chen, Shaohan Wu, Zhengwei Song, Jianguo Fei

**Affiliations:** Department of Surgery, The Second Affiliated Hospital of Jiaxing University, Jiaxing 314000, Zhejiang, China

## Abstract

**Background:**

To explore the clinical characteristics of reactivation of hepatitis B virus (HBV) in hepatocellular carcinoma (HCC) after transcatheter arterial chemoembolization (TACE). The pathological correlation of prognosis and hepatitis B virus reactivation has been given detailed analyses in our research.

**Methods:**

A total of 108 related TACE-treated HCC clinical data from January 2008 to January 2016 was gleaned and involved in this retrospective analysis. To lucubrate the nuance of survival rates between HBV reactivated group and HBV nonreactivated group, clinical data of each patient was analyzed in detail and refined the retrospective studies.

**Results:**

HBV reactivation occurred in 42 patients with a proportion of 38.9%. The detected HBV DNA level ≥10^4^ in patients showed a reactivation rate of 65.8% (25/38), which was significantly higher than the HBV DNA < 10^4^ cases (24.3%, 17/70). Research data revealed a conspicuous lower cellular immunity (*P* < 0.01) and better 2-year survival rate (*P*=0.03) in the HBV-reactivated group when compared to the nonreactivated group.

**Conclusion:**

Some of the patients with primary hepatocellular carcinoma possibly had HBV reactivation at post-TACE-therapy. And the predominant risk factors of HBV reactivation are positive HBV test and immunosuppression. Our study suggested that HBV reactivation at post-TACE-therapy is an independent predictor of poor prognosis and low survival rate as well as a crucial reason for poor prognosis and lower survival rate, which indirectly proved that it is urgent to necessitate the antiviral therapy and immune enhancer in improving the curative effect and prognosis of HCC patients.

## 1. Introduction

Hepatocellular carcinoma (HCC) is a common malignant cancer that perils human health, which is a serious cause of mortality [1]. Recently, the hepatitis B virus- (HBV-) or hepatitis C virus- (HCV-) related cirrhosis is recognized as the main reason that induces hepatocellular carcinoma (HCC) [2]. There is always a pathological correlation between HCC and HBV-related cirrhosis in China, which encourages us to continue the exploration work of whether HBV reactivation is an independent HCC mobility predictor, and the related clinical features were analyzed carefully to discover the relevance between HBV reactivation and the prognosis of HCC patients, as demonstrated in the pivotal paper that HBV-related cirrhosis is also an independent predictor of HCC and an independent predictor of death in subjects with HBV infection also in other countries [3]. Because of the high degree of malignancy and inconspicuous symptoms, most patients were diagnosed with liver cancer only in the late stage, and the radical resection rate was less than 30% [4]. Current reported studies declared that transcatheter arterial chemoembolization (TACE) is the first choice for unresectable and unresectable advanced liver cancer treatment [5–7], which significantly improved the survival rate of HCC patients [8, 9]. Nevertheless, some researchers showed that TACE therapy might impair liver function and triggered unfavorable prognosis via activating HBV [10, 11]. Since the exact cause of HBV reactivation and the pathological relation between HBV and HCC prognosis still remain under investigation, we retrospectively analyzed a total of 108 cases of HCC patients with TACE treatment to discover the influencing factors of HBV reactivation by TACE and its relationship with the prognosis of patients.

## 2. Patients and Methods

### 2.1. Patients

From January 2008 to January 2016, patients with hepatitis B-related liver cancer who underwent TACE in the Department of Hepatobiliary Surgery, the second affiliated hospital of Jiaxing University, were collected. Patients were enrolled in this study when meeting the following criteria: age ≥20 years; histological or clinical diagnosis of HCC by early tumor staining, dynamic CT, and dynamic magnetic resonance imaging (MRI); the presence of unresectable HCC; absence of indications for liver transplantation and local ablation (radiofrequency ablation, percutaneous ethanol injection, and microwave coagulation); Child-Pugh A or B; PS 0-2; and a more than 60-day lifespan. Exclusion criteria were as follows: receiving liver cancer treatment within 28 days before TACE; frequent use of phenytoin, warfarin, or flucytosine; severe heart failure; uncontrollable diabetes mellitus; active infection; women of childbearing age who did not use effective contraceptives; pregnancy or lactation; severe drugs' allergic; mental disorders; watery diarrhea; moderate or obvious thoracic cavity Effusion or ascites; and other serious diseases. All patients received TACE for the first time and followed the principle of TACE.

The study was approved by the institutional review committee of Jiaxing Second Hospital in Zhejiang Province. Written informed consent was obtained from each participant.

### 2.2. Treatment

Seldinger technology was utilized to conduct transcatheter arterial chemoembolization (TACE). The catheter was inserted into the common hepatic artery or the proper hepatic artery through the right inguinal femoral artery puncture. Splanchnic angiography was performed to assess tumor size, location, and arterial supply. The tip of the catheter was introduced into the tumor-supplying artery. Subsequently, 500–1000 mg of fluorouridine purchased from Zhejiang Haishun pharmaceutical, Zhejiang, China; 60 mg of epirubicin purchased from Pfizer, Bronx, New York, United States; 60 mg of cisplatin obtained from Qilu pharmaceutical, Shandong, China; and 10 ml of iodized oil obtained from GBE, Villepin, France, were injected into tumor blood vessels. Finally, we injected the gelatin sponge particles (Jinling Pharmaceutical, Jianshu, China) to embolize the blood supply to the tumor.

### 2.3. Data Collection, Definition, and Follow-Up

A retrospective analysis of all selected cases data was then performed using SPSS software. The levels of HBV DNA and alpha-fetoprotein (AFP) were measured before and 1 month after surgery. Flow cytometry was adapted to detect the preoperation and 1-week postoperation quantities of CD3+, CD4+, and CD8+ T cells. A patient was considered positive for HBV DNA when the HBV DNA level is over 1000 copies/mL. Serum HBV DNA levels 10 times higher than baseline are considered to be HBV reactivation [12]. All patients were followed up in outpatients or by telephone.

### 2.4. Statistical Analysis

Data was analyzed using SPSS (17th edition; IBM, Chicago, IL, United States). Continuous variation was compared by the *t*-test and partly by the chi-square test. Kaplan-Meier analysis was used for survival analysis. A two-sided *P* value <0.05 was considered statistically significant.

## 3. Results

### 3.1. Features of Enrolled Patients

A retrospective analysis of 108 patients was done, including 69 males and 39 females, and the average age was 58.69 ± 10.23 years (33–83 years). We divided 71 cases of Child-Pugh A and 37 cases of Child-Pugh B into two groups, of which 81 cases were single nodules and 27 cases were multiple nodules. The average size of the nodules was 6.72 ± 2.08 cm (3–11 cm). And there were twenty-six cases of portal vein cancer thrombosis comorbidity (PVTT).

### 3.2. Alteration of HBV DNA Level before and after TACE Therapy

Before TACE, patients with a proportion of 67.65% (73/108) were tested HBV DNA positive. One month after therapy of TACE, 42 patients had developed HBV reactivation (of which 24 cases of Child A and 18 cases of Child B), 3 patients who were tested HBV DNA negative subsequently turned into positive, and 39 patients had a 10-fold increase in HBV DNA levels. The reactivation of HBV has no distinct correlation with nodules number, the size of lesions, gender, age, HBeAg, liver cirrhosis, and PVTT. Compared with 76 HBV DNA positive patients (2.79 ± 2.68 × 10^4^ copies/mL) after TACE, the HBV DNA levels of 73 HBV DNA positive patients before TACE (2.44 ± 3.19 × 10^5^ copies/mL) were significantly increased (*P* < 0.01).

### 3.3. Changes of T Cells before and after TACE Therapy

In Table 1, the cell quantities of CD3+ and CD4+ were shown, and the proportion of CD4+/CD8+ in patients after TACE decreased more significantly than that before TACE (*P* < 0.01), which indicates that the patient's immune system is suppressed after TACE.

### 3.4. Affecting Factors of HBV Reactivation

Patients were allocated into two groups based on whether HBV reactivation occurred, and the clinical data of these two groups were compared to give an analysis. Patients with high HBV DNA levels (>10^4^ copies/mL) were more likely to develop HBV reactivation (65.79% [25/38]) than patients with low HBV DNA levels (<10^4^ copies/mL) (24.29% [17/70], *P* < 0.01) (Table 2). The immune function of patients with HBV reactivation was significantly reduced (*P* < 0.01). Nevertheless, other analytical results of related clinical features demonstrated that there were no statistical differences in age, gender, hepatitis B e antigen (HBeAg), child grade, cirrhosis comorbidity, and PVTT comorbidity between the two groups.

## 4. Survival

**Figure 1:**
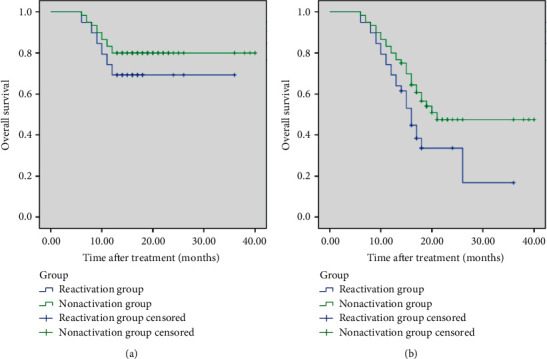
Survival curve of HBV-reactivated patients and HBV-nonreactivated patients. (a) Curve of one-year survival rate. (b) Curve of two-year survival rate.

Patients' follow-up was conducted in outpatients or by telephone with an average of 17.8-month of follow-up time (range: 3–40 months). 9 cases were lost, which accounted for 8.3%. There was no significant difference (*P* > 0.05) in one-year survival rate between patients with HBV reactivation (69.2%) and patients without HBV reactivation (80.0%, Figure 1(a)). However, as shown in Figure 1(b), patients with HBV reactivation (35.9%) showed a significantly lower 2-year survival rate when compared to those without HBV reactivation (53.3%) (*P*=0.03).

## 5. Discussion

TACE is recently recognized as the optimal treatment approach in curing unresectable intermediate- and advanced-stage HCC [13]. Most HCC patients have a comorbidity of HBV infection [14]. It has been reported that the immune response is suppressed after TACE; however, the immune balance between the host and HBV replication is disturbed, thereby reactivating HBV. As a result, patients again develop active hepatitis that aggravates liver damage [15, 16]. To prevent the onset of HBV reactivation after TACE, further liver protection is supposed to be performed for the patients who underwent TACE. With this approach, we can postpone the liver fibrosis development and improve the prognosis of patients. Nevertheless, the underlying mechanism through which HBV reactivation occurs after TACE still remains unknown.

In this study, the incidence of HBV reactivation of 108 HBV-related HCC patients who had received TACE was retrospectively analyzed, and the results indicate that 42 cases have further developed HBV positive with an incidence of 38.89%, of which 24 cases were Child A and 18 cases were Child B. In 3 cases, HBV negative was transformed into a positive state. Furthermore, in 39 cases, there was a tenfold enhancement in the HBV DNA level after TACE. Additionally, our research discovered that there was no conspicuous association between HBV reactivation and gender, age, Child-Pugh grade, HBeAg, liver cirrhosis, nodules number, and PVTT (Table 2). Previous studies have reported that HBeAg positive status and an intervention method are independent HBV reactivation risk factors after TACE [17]. Yet, our analytical result did not support this declaration, and we discovered that there is little significant difference in the HBeAg parameter with or without HBV reactivation. These data indicate that HBV reactivation occurs in some patients after undergoing TACE. Presently, there has been no consensus on the risk factors that reactivate HBV. Previous studies have reported that HBeAg positive status and an intervention method are independent HBV reactivation risk factors after TACE [17]. Our data indicates that the incidence of HBV reactivation is significantly higher in high HBV DNA level patients before TACE than in that low HBV DNA level patients before TACE. In patients with high HBV DNA levels before TACE, the liver function and HBV DNA level should be intensively monitored after TACE. As soon as HBV reactivation occurs, an antiviral therapy should immediately be provided to these patients. Hence, we can relieve the live damage caused by HBV reactivation in these patients. Our data also indicated that the quantities of CD3+ and CD4+ cells and the proportion of CD4+/CD8+ were conspicuously lower after TACE. Thus, the immune response was suppressed in patients after TACE. Some normal liver tissues may be damaged while performing the procedure of embolization. This factor may also contribute to immune suppression. Moreover, we have found that the immune response is suppressed more effectively in HBV reactivation patients after TACE. This might be because the immune suppression after TACE disturbs the immune balance in these patients, which facilitates HBV replication and reactivation. Finally, liver damage is intensified with HBV reactivation. We recommend that these patients should be provided with a combination of immunopotentiation therapy and antiviral therapy in an early phase of TACE therapy.

In this study, we have found that the AFP level of HBV reactivation patients is conspicuously higher than that in patients without HBV reactivation. Previous reports have indicated that the serum AFP level affects the outcomes of TACE in HCC patients, so the serum AFP level is an independent factor affecting survival time [18, 19]. Additionally, our analytical data demonstrated that there is no significant difference in the related clinical characteristics, including HBeAg, liver cirrhosis, the number of nodules, and PVTT (*P* > 0.05). Our results and previous reports have proved that HBV reactivation can affect the efficacy of TACE therapy. Moreover, our follow-up data indicated that the two-year survival rate in patients with HBV reactivation was conspicuously lower than that in patients without HBV reactivation. This might be because HBV is highly replicated in patients with HBV reactivation, causing damage to liver function and suppressing the immune response. These events facilitate the development of HCC, resulting in a poor prognosis of HBV reactivation patients. From the analytical results of this study, we consider that the reactivation of HBV would thereby aggregate the course of HCC patients, and antiviral therapy against HBV is of great importance and is well recommended for the prognosis improvement of HCC patients who received TACE treatment, which is consistent with the consequence declared by Granito and Bolondi [20].

In HCC patients who developed HBV, antiviral therapy is usually recommended by clinicians. A recent study shows that antiviral therapy can improve survival time for patients with HBV-related hepatocellular carcinomas [21]. Nagamatsu et al. [22] have reported that HBV DNA level had decreased in 17 HCC patients with HBV development and TACE; moreover, the HBV DNA level did not rebound in these patients. Furthermore, no liver damage was observed during TACE therapy in 8 patients administering lamivudine (100 mg) once per day. On the other hand, of 9 patients without antiviral therapy, 6 patients experienced aggravated liver damage. This data indicates that antiviral therapy can decrease HBV DNA level and prevent aggravated liver damage in HBV-related HCC patients undergoing TACE therapy. A prospective study on 73 HCC patients undergoing TACE conducted by Jang et al. [23] indicates that prophylaxis along with lamivudine can decrease the incidence of HBV reactivation in patients who have undergone TACE therapy. However, several studies have reported that the incidence of HBV reactivation was still 20% in HCC patients who had been treated with both TACE and antiviral therapies [24, 25]. This might because the virus undergoes mutation when the administration of antiviral therapy is delayed. With the long-term use of lamivudine, the proportion of drug-resistant viral mutations increased. An increase in mutations is always associated with higher levels of HBV DNA and ALT [26]. Recently, it has been reported that when lamivudine is replaced with entecavir or tenofovir, there is better efficacy in suppressing HBV DNA levels. However, the drug-resistant viral mutations dominate during prophylaxis in HBV-related HCC patients undergoing chemotherapy [27]. This experience might work in HCC patients undergoing TACE; however, further studies are needed to confirm it.

In conclusion, not all HBV-related HCC patients experience HBV reactivation after TACE. HBV reactivation is more likely to happen in patients when HBV DNA level >10^4^ copies/mL. Therefore, early prophylaxis should be provided by administering a combination of antiviral drugs and immunopotentiation therapy. Further studies are needed to confirm the efficacy of this combinatorial prophylaxis on the prognosis and survival time of patients with HBV-related HCC.

## Figures and Tables

**Table 1:** T cell changes in patients before and after TACE.

T cell subgroups	Pre-TACE	Post-TACE	*t* value	*P* value
CD3+ (%)	55.36 ± 8.76	50.65 ± 5.97	4.718	<0.001
CD4+ (%)	26.79 ± 4.31	22.77 ± 3.63	7.335	<0.001
CD4+/CD8+	0.95 ± 0.13	0.83 ± 0.17	5.828	<0.001

**Table 2:** Characteristics and changes in patients with HBV reactivation and those without HBV reactivation.

Characteristics	HBV reactivation (*n* = 42)	No reactivation (*n* = 66)	*t* or *χ*^2^	*P* value
Gender			0.230	0.632
Male	28	41		
Female	14	25		
Age (years)	57.40 ± 9.59	59.51 ± 10.6	−1.046	0.298
Child-Pugh grade			2.256	0.133
A	24	47		
B	18	19		
HBeAg			2.494	0.114
Positive	25	29		
Negative	17	37		
Liver cirrhosis			2.693	0.101
Yes	20	42		
No	22	24		
HBV DNA before TACE (copies/mL)			17.852	<0.001
≥10^4^	25	13		
<10^4^	17	53		
Number of nodules			1.299	0.255
Single	29	52		
Multiple	13	14		
Tumor diameter (cm)	6.83 ± 2.12	6.65 ± 2.07	0.441	0.660
PVTT			0.003	0.959
Yes	10	16		
No	32	50		
AFP before TACE (ng/mL)	463.78 ± 251.57	430.44 ± 254.21	0.667	0.506
AFP after TACE (ng/mL)	333.90 ± 200.31	244.89 ± 174.47	2.435	0.017
T cells before TACE				
CD3+ (%)	55.77 ± 9.72	55.10 ± 8.17	0.387	0.700
CD4+ (%)	26.82 ± 4.63	26.76 ± 4.12	0.068	0.946
CD4+/CD8+	0.94 ± 0.11	0.96 ± 0.14	–0.905	0.367
T cells after TACE				
CD3+ (%)	48.57 ± 6.28	51.97 ± 5.40	−2.992	0.003
CD4+ (%)	20.91 ± 3.33	23.95 ± 3.30	−4.638	<0.001
CD4+/CD8+	0.72 ± 0.11	0.86 ± 0.13	−2.393	0.018

AFP, alpha-fetoprotein; PVTT, portal vein tumor thrombus; TACE, transcatheter arterial chemoembolization; HBeAg, hepatitis B e antigen.

## Data Availability

The data in this study were collected from the Second Affiliated Hospital of Jiaxing University.

## Abbreviation

HBV: Hepatitis B virus
HCV: Hepatitis C virus
HCC: Hepatocellular carcinoma
TACE: Transcatheter arterial chemoembolization
CT: Computed tomography
MRI: Magnetic resonance imaging.
